# Supplementary material for: Transcriptome-Wide Identification of CCCH-Type Zinc Finger Proteins Family in Pinus massoniana and RR-TZF Proteins in Stress Response
Source: Genes (Basel). 2022 Sep 13;13(9):1639. doi: 10.3390/genes13091639 (PMC9498899; doi:10.3390/genes13091639)
Supplement: Supplementary file 1 [file genes-13-01639-s001.zip › Figures S1&sS2.pdf]

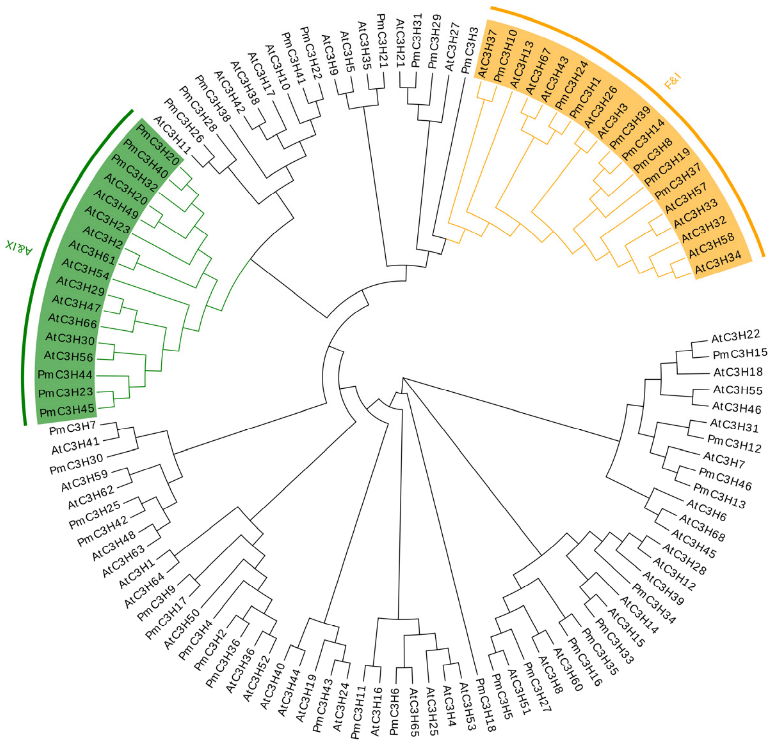

Figure S1: Phylogenetic analysis of CCCH proteins in *P. massoniana* and *Arabidopsis thaliana,* "A& IX": Group A in *P. massoniana* and subfamily IX in *Arabidopsis thaliana;* "F&I": Group F in *P. massoniana* and subfamily I in *Arabidopsis thaliana*

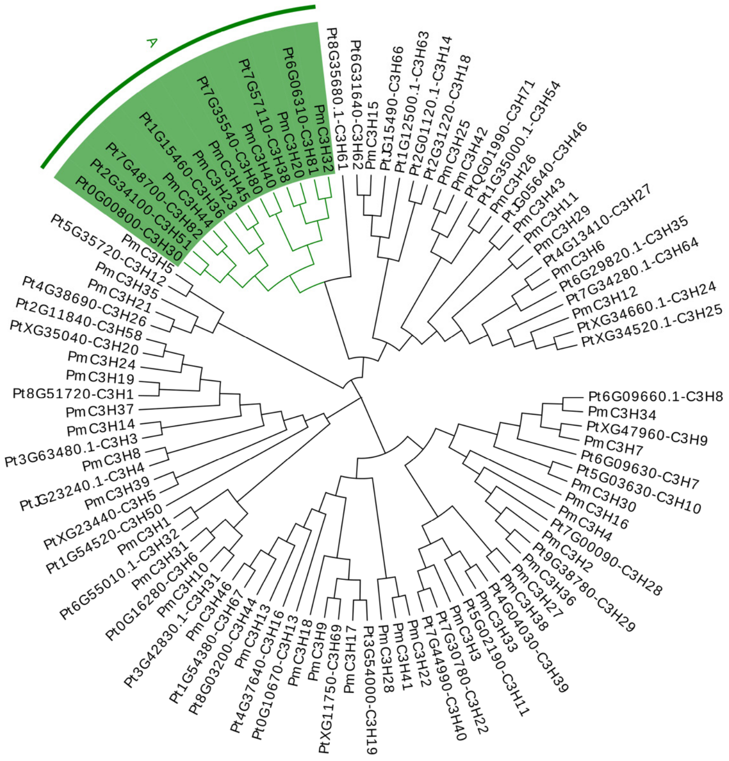

Figure S2: Phylogenetic analysis of CCCH proteins in *P. massoniana* and *Pinus tabulaeformis.* Group A cantains 6 PmRR-TZFs and 7 PtaRR-TZF genes.
